# Influence of the coexistence of visual impairment, hearing impairment, and masticatory discomfort on the quality of life of middle-aged adults: an analysis based on the 2019 and 2020 Korea National Health and Nutrition Examination Survey

**DOI:** 10.7586/jkbns.24.009

**Published:** 2024-05-28

**Authors:** Jeong-Eun Kim, Yun-Hee Kim

**Affiliations:** Department of Nursing, Pukyong National University, Busan, Korea

**Keywords:** Quality of life, Vision disorders, Hearing, Mastication

## Abstract

**Purpose:**

Visual impairment, hearing impairment, and masticatory discomfort each influence quality of life (QoL). However, little is known regarding the impact of their coexistence on QoL. Therefore, we aimed to investigate the influence of the coexistence of visual impairment, hearing impairment, and masticatory discomfort on QoL among middle-aged adults aged 40-64.

**Methods:**

This study involved a secondary data analysis utilizing the data from years 1 and 2 of the eighth Korea National Health and Nutrition Examination Survey. To evaluate the influence of the coexistence of visual impairment, hearing impairment, and masticatory discomfort on QoL, we conducted a complex sample hierarchical multiple regression analysis.

**Results:**

When visual impairment, hearing impairment, and masticatory discomfort coexisted, the QoL was significantly lower than in individuals without any of these conditions.

**Conclusion:**

The coexistence of visual impairment, hearing impairment, and masticatory discomfort was negatively correlated with QoL. Therefore, it is important to prepare for old age through appropriate health management during middle age.

## INTRODUCTION

Globally, the population is aging rapidly [1]. In 2023, the elderly population in South Korea accounted for 18.4% of the total population, showing a rapid pace of aging [2]. In 2022, the number of elderly individuals aged 65 and over covered by health insurance was 8.75 million, with medical expenses for the elderly totaling 45.7 trillion 647 billion won, which is a 1.4-fold increase compared to 2018, indicating an increasing social and national burden [3]. Therefore, preparation for preventing and managing health issues from middle age onwards is necessary for a stable retirement. However, due to experiencing declines in physical, mental, and biological capacities and health, improper management of middle age can lead to significant challenges in old age [4]. Therefore, preventive care is crucial for preparing for life in old age, and various strategies need to be developed to enhance the quality of life (QoL) in middle age.

Aging is a universal process that all humans undergo throughout their growth and development. When aging leads to visual impairment, hearing impairment, or cognitive decline, it can significantly influence QoL [5-7]. Vision, being one of the most critical sensory functions of the human body, when impaired, can negatively affect an individual's daily life and may lead to functional disabilities and other health issues [8]. Visual impairment affects work performance, mobility, and contributes to depression and anxiety, with more severe visual impairment leading to a greater reduction in QoL [5,9]. Presbyopia, which occurs due to a decrease in the ability to adjust focus, often first manifests in the early 40s, leading to the alternative term 'middle-aged vision [10].' These symptoms of presbyopia during middle age can significantly impede daily activities, occasionally diminishing work productivity and overall life contentment. In the United States, the prevalence of visual impairment is 17% among adults aged 45 and older, rising to 26.5% among those aged 75 and older [11]. Aging is a prominent risk factor for visual impairment, with a higher incidence among the elderly compared to middle-aged adults. Despite this, the influence of visual impairment on QoL among middle-aged adults should not be underestimated, underscoring the need for targeted management and intervention.

People experiencing hearing impairment negative influences in both physical and mental domains. According to a study utilizing census data from the UK, it was reported that one out of every ten middle-aged individuals suffers from significant hearing impairment, with the prevalence increasing particularly with advancing age, especially after the mid-50s [12]. Hearing impairment is associated with an increased risk of dementia [13], depression, higher levels of stress, somatization, loneliness, and a negative influence on QoL [6,14,15]. This is increasingly considered a significant public health issue, highlighting the growing need for hearing care management among the middle-aged population.

People with low mastication ability are associated with dementia and Alzheimer's disease [16], which leads to a lower QoL and decreased survival rates [17,18]. Based on a study investigating masticatory discomfort according to types of dental prostheses using the Korea National Health and Nutrition Examination Survey (KNHANES), the incidence of discomfort stood at 14.2% among those in their 40s, showing a marked rise among individuals in their 50s. Additionally, the proportion of those experiencing masticatory discomfort increased with age [19]. Masticatory ability influences the enjoyment of meals and diet, and it is also linked to QoL. Hence, it is crucial to explore the connection between the QoL in middle age and issues such as visual and hearing impairments, as well as masticatory discomfort.

Coexistence of sensory impairments negatively influences depression, loneliness, social isolation, self-esteem, and autonomy, leading to reduced quality in physical, mental, and social functions [20,21]. Dual sensory impairment of visual and hearing in middle-aged individuals increased ninefold among those in the oldest age group, 65–69 years, compared to those in the 40–44 years age group. Additionally, visual and hearing impairments often occur concurrently, and when both sensory issues are present, the age-related proportional increase is greater than when either visual or hearing impairment is experienced independently [22].

Dental health can also affect hearing. Sound stimuli through the teeth and jaw are transmitted to the cochlea via bone conduction, a phenomenon known as "dentauralhearing [23]." When a sound source is applied to the skull, vibrations can pass through it due to the skull's fibrous-elastic properties. It is unclear how this mechanical energy affects the ear sac, middle ear structures, and external auditory canal [24]. People who have lost more than half of their teeth are 1.64 times more likely to experience hearing impairment compared to those who have not [25]. Additionally, in a study evaluating hearing after providing implant-supported complete dentures to completely edentulous individuals, it was reported that implants improved bone conduction similar to teeth, enhancing hearing 1 month after implant placement [26]. Furthermore, individuals without occlusal vertical dimension, which is the vertical distance when the teeth of the maxilla and mandible come into contact, showed higher rates of hearing impairment compared to those with occlusal vertical dimension [27]. Based on these studies confirming the association between dental and hearing health, it is necessary to consider sensory impairments and masticatory function together.

Individuals with masticatory issues and comorbidities are more likely to require dental treatment compared to those without these conditions [28]. When multiple tooth loss and masticatory dysfunction occur, the likelihood of cognitive impairment is significantly higher [15]. If malocclusion and temporomandibular joint disorders accompany these issues, masticatory dysfunction becomes more pronounced, negatively influencing oral health-related QoL [29]. Such sensory and masticatory function impairments have long-term effects on the QoL, leading to a deterioration in physical, emotional, and social functioning over the years [21]. With the aging demographic trend persisting, age-related diseases will become a growing concern not only domestically but also globally for decades to come.

However, research on sensory and functional impairments has mainly focused on individuals aged 65 and above due to rapid aging, with limited and insufficient studies targeting the middle-aged population in the transitional period to old age. While several previous studies have explored the influence of dual sensory impairment, encompassing both visual and hearing impairments, on QoL, it was challenging to find research specifically examining the effects of three or more coexisting issues such as masticatory discomfort on QoL.

This study aims to understand the influence of coexisting visual impairment, hearing impairment, and masticatory discomfort on the QoL during middle age, with the goal of highlighting the importance of health management and providing foundational data for developing effective nursing practices to facilitate a healthy transition from middle age to old age.

## METHODS

### 1. Research design

This study involves a secondary data analysis utilizing the 8th KNHANES 1st and 2nd year data to investigate the influence of coexisting visual impairment, hearing impairment, and masticatory discomfort on the QoL in middle-aged individuals.

### 2. Participants

**Figure 1.**
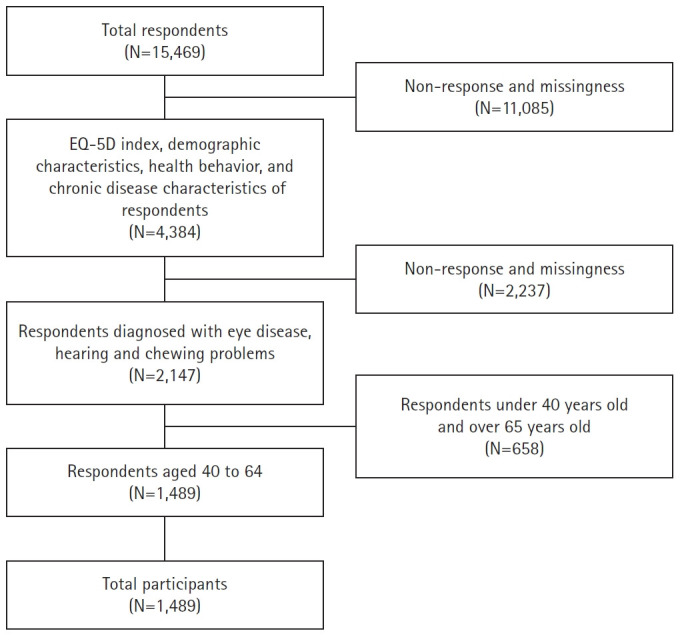
Flow chart of the study protocol.

This study integrated the 1st (2019) and 2nd (2020) data from the 8th KNHANES, utilizing health surveys and health examinations. Out of the 3-year data, only the two years containing both the independent and dependent variables were used. The 1st and 2nd year surveys of the 8th KNHANES employed a rotating panel design, with the survey district As the primary sampling unit with households serving as the secondary sampling unit. The total sample size was 372 survey districts and 10,975 households, with a total of 15,469 participants. This study targeted 1,489 participants aged 40 to 64 who responded to questions regarding the QoL index (EQ-5D index), diagnosis of ocular diseases, self-reported hearing status, and chewing problems (Figure 1).

### 3. Research instrument

#### 1) Demographic characteristics, health behavior and chronic disease-related characteristics

The demographic characteristics of the participants were examined based on sex and educational level. Education level is divided into high school graduate or above, and less than middle school graduate.

In categorizing health behaviors, the drinking frequency was classified as follows: drinking 4 or more times a week and approximately 2-3 times a week were categorized as 'less than 4 times a month.' Drinking about 2-4 times a month, roughly once per month, and less than once a month were categorized as '2 or more times a week'. Abstaining from alcohol consumption entirely in the past year was classified as 'non-drinker'. Smoking status was divided between smokers and non-smokers. Physical activity status was determined using the aerobic physical activity prevalence rate, wherein engaging in moderate-intensity physical activity for at least 2 hours and 30 minutes per week, high-intensity physical activity for at least 1 hour and 15 minutes per week, or a combination of both, equivalent to the respective durations, was categorized as 'exercise'. Conversely, the absence of such activity was classified as 'non-exercise'. In terms of obesity prevalence, pre-obesity and stages 1, 2, and 3 obesity were grouped together as 'obesity', while individuals with normal weight and those classified as underweight were categorized as 'non-obesity.'

Characteristics related to chronic diseases were divided into 'yes' or 'no' based on whether hypertension, dyslipidemia, stroke, cardiovascular disease, and diabetes had been diagnosed by a doctor.

#### 2) Visual impairment, hearing impairment, masticatory discomfort

Visual impairment was determined based on the diagnosis of ophthalmic conditions such as glaucoma, cataracts, age-related macular degeneration, retinal vascular occlusion, diabetic retinopathy, dry eye syndrome, or other eye diseases. If diagnosed with any of these eye conditions, the individual was classified as 'yes,' while those not diagnosed were classified as 'no.' Hearing impairment was determined using self-reported hearing questions, with 'completely unable to hear,' 'very uncomfortable,' and 'slightly uncomfortable' categorized as 'yes,' while 'not uncomfortable' was categorized as 'no.' Masticatory discomfort was assessed using questions about chewing problems, with 'very uncomfortable' and 'uncomfortable' categorized as 'yes,' while 'so-so,' 'not uncomfortable,' and 'not uncomfortable at all' were categorized as 'no.'

To identify the coexistence of visual impairment, hearing impairment, and masticatory discomfort, we categorized them into A, B, and C. A represents cases with only one of the conditions (visual impairment only, hearing impairment only, masticatory discomfort only). B includes cases with two of the conditions (visual impairment and hearing impairment, visual impairment and masticatory discomfort, hearing impairment and masticatory discomfort). C comprises cases with all three conditions (visual impairment, hearing impairment, and masticatory discomfort). In this way, we divided them into a total of seven categories, with individuals without any of the three conditions serving as the reference group.

#### 3) QoL

The QoL tool utilized the EQ-5D index, which measures health status on a scale ranging from the worst health state －1 to the best health state +1 using a single number. It evaluates five facets: mobility, self-care, typical activities, pain/discomfort, and anxiety/depression. Each facet is assessed on three tiers: absence of issues, moderate challenges, and severe difficulties.

The EQ-5D index in this study was obtained from a quality weight estimation study of the EQ-5D QoL tool published by the Korea Disease Control and Prevention Agency (KDCA) [30]. To calculate the EQ-5D value for Koreans, the following weighting formula was applied: EQ-5D = 1 － [0.05 + 0.096(M2) + 0.418(M3) + 0.046(SC2) + 0.136(SC3) + 0.051(UA2) + 0.208(UA3) + 0.037(PD2) + 0.151(PD3) + 0.043(AD2) + 0.158(AD3) + 0.05(N3)]. When all five items have a score of 1, the EQ-5D value is 1, and when all five items have a score of 3, indicating the worst health state, the value is －0.17. A higher EQ-5D index score indicates a higher perceived QoL.

### 4. Data analysis

Statistical analysis was performed with SPSS/WIN version 29.0.1.0 (IBM Corp., Armonk, NY, USA), using complex sample analysis with stratification, clustering, and weighting. Demographic characteristics, health behaviors, and chronic disease-related characteristics were analyzed using complex sample frequency analysis and descriptive statistics to get frequency, percentage, mean, and standard error. QoL differences based on subject characteristics were analyzed using t-tests and one-way ANOVA, with post-hoc analysis using Bonferroni correction. QoL differences from visual and hearing impairments and masticatory discomfort, individually and in combination, were analyzed using complex sample frequency analysis, descriptive statistics, and t-tests. A hierarchical multiple regression analysis was used to study how visual and hearing impairments, and masticatory discomfort affect QoL in middle-aged adults. Model 1 had no controls, model 2 controlled for demographics, model 3 added health behaviors, and model 4 included chronic disease-related characteristics.

### 5. Ethical considerations

This study obtained endorsement from the research ethics committee of the affiliated university (1041386-202403-HR-41-02) and obtained authorization for data usage by adhering to compliance and security protocols. It followed the procedures outlined by the KDCA for accessing raw data from the KNHANES. KNHANES was conducted following approval from the research ethics review committee of the KDCA.

In compliance with the personal data protection act and the statistical law, the data were anonymized to ensure that individuals could not be identified from the survey data, thus ensuring the anonymity and confidentiality of the participants.

## RESULTS

### 1. Difference in QoL based on demographic characteristics, health behaviors, and chronic disease-related characteristics

Among the 1,489 participants, 1,270 (89.0%) were male, and 219 (11.0%) were female. The QoL was lower in females than in males (*p* = .011). Additionally, 1,237 (86.0%) had an education level of high school graduate or above, while 252 (14.0%) had an education level of junior high school graduate or below. It has been shown that those with less than a middle school education have lower QoL compared to those with a high school diploma or higher (*p* < .001).

The largest group by drinking frequency had 676 people (44.9%) drinking fewer than four times a month, followed by 613 people (42.5%) who drank twice a week or more, and 200 people (12.5%) who were non-drinkers. QoL was highest for those who drank twice a week or more, followed by those drinking fewer than four times a month, then non-drinkers (*p* = .027). However, post-hoc analysis showed no significant differences. The smoking group consisted of 655 individuals (44.0%), while the non-smoking group consisted of 834 individuals (56.0%). The exercise group comprised 624 individuals (42.6%), while the non-exercise group comprised 865 individuals (57.4%). The obesity group included 1,047 individuals (72.8%), whereas the non-obesity group included 442 individuals (27.2%). The QoL was higher in the obesity group compared to the non-obesity group (*p* = .019).

In terms of chronic disease-related characteristics, the diagnosed group for hypertension comprised 400 individuals (26.1%), while the undiagnosed group consisted of 1,089 individuals (73.9%). For dyslipidemia, the diagnosed group included 353 individuals (23.0%), whereas the undiagnosed group comprised 1,136 individuals (77.0%). In the case of stroke, 23 individuals (1.6%) were diagnosed, while 1,466 individuals (98.4%) were undiagnosed. Regarding cardiovascular diseases, there were 44 individuals (2.8%) in the diagnosed group and 1,445 individuals (97.2%) in the undiagnosed group, with no significant difference observed in QoL. The diagnosed group for diabetes included 165 individuals (11.0%), while the undiagnosed group comprised 1,324 individuals (89.0%). The QoL was lower in the diagnosed group for diabetes compared to the undiagnosed group (*p* = .006).

Among the participants, 271 (17.2%) had visual impairment, while 1,218 (82.8%) did not; 218 (13.5%) had hearing impairment, while 1,271 (86.5%) did not; and 361 (23.1%) had masticatory discomfort, while 1,128 (76.9%) did not. Those with visual impairment, hearing impairment, and masticatory discomfort showed lower QoL compared to those without each respective condition (*p* = .007, *p* < .001, *p* < .001). Furthermore, 682 (45.8%) had at least one of these conditions, whereas 807 (54.2%) had none. Those with at least one of the conditions exhibited lower QoL compared to those with none (*p* < .001) (Table 1).

### 2. Difference in QoL based on the coexistence of visual impairment, hearing impairment, and masticatory discomfort

The differences in QoL when visual impairment, hearing impairment, and masticatory discomfort coexist were analyzed among 682 individuals with at least one of these conditions: visual impairment, hearing imapairment, or masticatory discomfort. They were categorized into groups A, B, and C, based on the number of coexisting impairments among the three. Comparing to the reference group of 807 individuals with none of the three conditions, in category A, there were 169 individuals (16.3%) with visual impairment only, 123 individuals (12.7%) with hearing impairment only, and 238 individuals (22.5%) with masticatory discomfort only. It was discovered that individuals experiencing only masticatory discomfort exhibited a notably lower QoL compared to the reference group (*p* = .035). In category B, there were 29 individuals (3.3%) with both visual impairment and hearing impairment, 57 individuals (5.9%) with both visual impairment and masticatory discomfort, and 50 individuals (4.3%) with both hearing impairment and masticatory discomfort. In category B, all groups showed substantially inferior QoL compared to the reference group (*p* = .009, *p* = .009, *p* = .047). In category C, which included individuals with all three issues, there were 16 individuals (1.5%). They showed significantly lower QoL compared to the reference group (*p* = .008) (Table 2).

### 3. Influence of the coexistence of visual impairment, hearing impairment, and masticatory discomfort on QoL

To investigate the influence of the coexistence of visual impairment, hearing impairment, and masticatory discomfort on QoL, we conducted hierarchical multiple regression analysis. This study analyzed the data by incorporating as control variables all factors that affect the QoL in the study subjects, including sex, education level, drinking frequency, obesity, and diabetes diagnosis, as well as smoking, physical activity, hypertension, cerebrovascular disease, and heart disease, in accordance with prior research findings [31-33]. Model 1 did not include any control variables, Model 2 added demographic factors, Model 3 included controls for health behaviors, and Model 4 added controls for chronic disease-related factors to examine the influence of categories A, B, and C on QoL. The results showed that Model 1 had an explanatory power of 3.0%, Model 2 had 5.6%, Model 3 had 7.3%, and Model 4 had 8.8%. Testing for multicollinearity among the independent variables revealed that the variance inflation factors for all independent variables ranged from 1.039 to 1.320, well below 10, and the tolerance values ranged from 0.757 to 0.966, all exceeding the minimum threshold of 0.1. These results indicate that there were no multicollinearity issues.

In the regression analysis results, when only one of visual impairment, hearing impairment, or masticatory discomfort was present (A), none of them showed a significant difference in QoL. In the case where two out of the three coexist (B), there was no significant difference when hearing impairment and masticatory discomfort were present (*p* = .276). However, when visual impairment and masticatory discomfort were present (*p* = .014) and when visual impairment and hearing impairment were present (*p* = .009), they significantly influenced QoL. Furthermore, when all three issues were present (C), it significantly influenced QoL (*p* = .022). While in some cases neither A nor B individually showed significant results, the presence of two or three coexisting issues tended to increase their influence compared to the presence of only one (Table 3).

## DISCUSSION

This study sought to explore the imfluence of the coexistence of visual impairment, hearing impairment, and masticatory discomfort on the QoL among adults aged 40 to 64 using the raw data from the 8th KNHANES conducted in 2019 and 2020.

In this study, a diminished QoL was observed among women, those with lower educational levels, non-drinkers, and non-obesity individuals, as well as those with diabetes. A study investigating factors influencing QoL among middle-aged individuals also found that general characteristics and health-related behaviors such as sex, educational level, drinking frequency, and obesity status significantly affected QoL [32]. Among characteristics related to chronic conditions, although measurement tools varied across study targeting adults, those with diabetes complications consistently exhibited lower QoL [34]. Furthermore, a study investigating the prevalence of chronic diseases among adults under 64 according to their QoL found a significantly higher prevalence of diabetes among those with lower QoL [33], which aligns with the results of this study. A study measuring QoL among adults found that as age increases from the 30s to the 40s and beyond, QoL tends to decline [35]. Therefore, it is important to manage the QoL during middle age to prepare for old age, emphasizing the need for strategies to address health-risk behaviors and disease prevention during middle age.

The results of this study indicate that visual impairment, hearing impairment, and masticatory discomfort each had a significant influence on QoL when present individually. A previous study, utilizing the KNHANES to measure vision among adults aged 19 and above, found that visual impairment and ocular diseases had an influence on activity limitation and QoL [36]. In a previous study focusing on middle-aged individuals using self-report measures, hearing impairment was found to significantly impair QoL [12]. Additionally, a study on the association between oral health status and QoL among adults under 64 found that masticatory discomfort had an influence on QoL [37], aligning with the findings of our study.

However, in this study, among participants with coexisting visual and hearing impairments and masticatory discomfort, those with only visual impairment, only hearing impairment, or only masticatory discomfort (Category A) did not show a significant influence on QoL. This difference is likely due to variances in the analysis methods between previous studies and our study. In previous studies, respondents with single occurrences of each independent variable might have been mixed with those who responded to other variables as well. However, in our study, individuals with only one of the three main independent variables—visual impairment only, hearing impairment only, or masticatory discomfort only—did not include respondents who answered to other variables. Furthermore, since our study focused on middle-aged individuals and had a limited number of participants in the categories of visual impairment only, hearing impairment only, and masticatory discomfort only among the coexistence states, this might have contributed to the findings. Therefore, it is suggested that future research, using not only the raw data from the 2019 and 2020 KNHANES but also a larger sample, is needed. Furthermore, the measurement tools for variables in our study were self-report measures, whereas previous studies utilized objective measurement tools, leading to methodological differences. Therefore, future research utilizing the same measurement tools is necessary for comparability.

Among the coexistence of two variables from visual impairment, hearing impairment, and masticatory discomfort (Category B), only visual impairment and hearing impairment, as well as visual impairment and masticatory discomfort, had a significant influence on QoL. This is consistent with previous studies showing that when visual and hearing impairments coexist, the QoL is lower than when each condition occurs alone [23]. Both visual and hearing impairments strongly affect subsequent functions such as physical function, mental health, and social function. However, visual impairment has a broader influence on functional status compared to hearing impairment [38]. Thus, while managing hearing impairment and masticatory discomfort is important, the management of visual impairment is of even greater significance due to its more extensive influence on daily life. The coexistence of both hearing impairment and masticatory discomfort did not exert a significant influence on QoL. Previous studies have reported an association between dental conditions and hearing [25-27,39], but research on the coexistence of masticatory function and hearing impairment is rare. Further investigations are required to examine the influence of the coexistence of hearing and masticatory function on QoL.

Finally, when all three conditions of visual impairment, hearing impairment, and masticatory discomfort coexisted (Category C), the QoL was significantly lower compared to individuals without all three conditions. Since vision allows people to detect clues about their environment and plan physical movements, a decline in visual acuity can lead to reduced ability to detect environmental hazards, potentially resulting in a decrease in physical functionality [40,41]. Similarly, hearing impairment increases the effort required to understand speech, altering neural activity in key auditory processing areas, which affects cognitive functions and may lead to impairments in memory and activities of daily living [42]. Issues with the primary function of teeth—mastication—can lead to decreased secretion of digestive enzymes, resulting in digestive problems. It can impair an individual's ability to eat, speak, socialize, and perform various everyday activities [7,43]. It is known from previous studies that while the visual and hearing impairments, as well as masticatory discomfort [20-22,29], each have a negative influence on an individual's QoL, the QoL is even lower when two or more of these issues coexist. Research on the influence on QoL when three factors—visual and hearing impairments, and masticatory discomfort—coexist is scarce. However, the results of this study confirm that when these issues are present together, they affect basic living activities, leading to a decline in QoL. If these impairments coexist in middle age, it can be assumed that they may persist into old age, with a cumulative influence on reducing QoL. The significance of this study lies in its confirmation that the coexistence of age-related sensory and functional impairments has a significantly greater influence on QoL than when they exist in isolation. This highlights the need to promote awareness of preventive healthcare for middle-aged individuals, as they are at a critical stage preceding old age. As aging progresses, visual and hearing impairments, and masticatory discomfort are often viewed as natural occurrences, leading to a lack of attention to potential comorbidities when interacting with patients in clinical nursing practice. In clinical nursing practice, it's essential to consider whether visual impairment, hearing impairment, and masticatory discomfort are co-occurring, rather than addressing them individually when assessing patients. This approach is crucial because when assessing patients, those with coexisting visual impairment, hearing impairment, and masticatory discomfort typically have lower QoL compared to those without such co-occurrences. This necessitates more attentive nursing care. By educating junior practitioners about this approach and implementing it in clinical nursing practice, the quality of nursing care can be improved, which is expected to lead to more effective health management for patients. In the midst of the human life cycle, it's crucial for those in middle age, transitioning between life stages and nearing old age, to prevent the coexistence of health conditions and prioritize consistent healthcare management. The prevalence of visual impairment, hearing impairment, and masticatory discomfort increases with age, influencing QoL. Middle-aged individuals experiencing sensory and functional impairments will continue to face quality-of-life challenges as they age. Given this, it's crucial to focus on health management at both personal and national levels to address these ongoing influences. On a personal level, maintaining healthy habits and regular physical activity are essential. On a national level, it's important to focus efforts on education, promotion, and support for early detection and prevention of coexisting health issues through initiatives such as health screenings.

This study aimed to investigate the influence of coexistence of visual impairment, hearing impairment, and masticatory discomfort on QoL. Nevertheless, there are certain constraints to take into account. Firstly, the sample size was limited as the study only focused on middle-aged individuals using raw data from 2019 and 2020, which may restrict the generalizability of the findings. Secondly, the measurement methods for visual impairment, hearing impairment, and masticatory discomfort tools involve a mixture of disease diagnosis and self-reporting, potentially reducing objectivity.

## CONCLUSION

This study examined how the coexistence of visual impairment, hearing impairment, and masticatory discomfort influences the QoL in middle-aged individuals, using raw data from the 8th KNHANES 2019, 2020. When these conditions existed singly, they did not significantly affect the QoL compared to individuals without any of the three conditions. However, when two conditions coexisted, only visual impairment with hearing impairment, and visual impairment with masticatory discomfort had an effect on QoL. Individuals with all three conditions experienced a significantly lower QoL.

The significance of this study lies in demonstrating that when visual impairment, hearing impairment, and masticatory discomfort—commonly seen as natural processes of aging—coexist, they influence the QoL in middle-aged individuals. Therefore, proper health management during middle age is crucial for a healthy transition into old age, necessitating both personal and national support to enhance the QoL.

Drawing from the conclusions of this study, the following suggestions are put forward. Conduct more research on how combined sensory and masticatory function impairments influence QoL, focusing on middle-aged individuals with a larger sample size. Use objective assessment tools to evaluate key variables for improved accuracy in future studies. Investigate how hearing impairment and masticatory discomfort affect QoL.

## Figures and Tables

**Table 1.** Differences in QoL based on Demographic Characteristics, Health Behaviors, and Chronic Disease-related Characteristics (N = 1,489)

Variables		n (%)	M ± SE	t or F (*p*)
Sex	Male	1,270 (89.0)	0.97 ± 0.00	2.55 (.011)
	Female	219 (11.0)	0.96 ± 0.01	
Education level	≥ High school	1,237 (86.0)	0.97 ± 0.00	4.04 (< .001)
	≤ Middle school	252 (14.0)	0.94 ± 0.01	
Drinking frequency	≥ 2 times a week^a^	613 (41.2)	0.97 ± 0.00	2.22 (.027)^†^
	< 4 times a month^b^	676 (45.4)	0.97 ± 0.00	
	Non-drinking^c^	200 (13.4)	0.96 ± 0.01	
Smoking status	Smoking	655 (44.0)	0.96 ± 0.00	-1.69 (.093)
	Non-smoking	834 (56.0)	0.97 ± 0.00	
Physical activity	Exercise	624 (42.6)	0.97 ± 0.00	0.96 (.337)
	Non-exercise	865 (57.4)	0.97 ± 0.00	
Obesity status	Obesity	1,047 (72.8)	0.97 ± 0.00	2.36 (.019)
	Non-obesity	442 (27.2)	0.96 ± 0.01	
Hypertension	Yes	400 (26.1)	0.97 ± 0.00	-0.39 (.695)
	No	1,089 (73.9)	0.97 ± 0.00	
Dyslipidemia	Yes	353 (23.0)	0.97 ± 0.00	-0.23 (.819)
	No	1,136 (77.0)	0.97 ± 0.00	
Stroke	Yes	23 (1.6)	0.94 ± 0.02	-1.27 (.204)
	No	1,466 (98.4)	0.97 ± 0.00	
Cardiovascular disease	Yes	44 (2.8)	0.95 ± 0.02	-1.04 (.300)
	No	1,445 (97.2)	0.97 ± 0.00	
Diabetes mellitus	Yes	165 (11.0)	0.95 ± 0.01	-2.79 (.006)
	No	1,324 (89.0)	0.97 ± 0.00	
VI	Yes	271 (17.2)	0.95 ± 0.01	-2.73 (.007)
	No	1,218 (82.8)	0.97 ± 0.00	
HI	Yes	218 (13.5)	0.95 ± 0.01	-3.44 (< .001)
	No	1,271 (86.5)	0.97 ± 0.00	
MD	Yes	361 (23.1)	0.95 ± 0.01	-3.64 (< .001)
	No	1,128 (76.9)	0.97 ± 0.00	
VI, HI, or MD	Yes	682 (45.8)	0.96 ± 0.00	-3.77 (< .001)
	No	807 (54.2)	0.98 ± 0.00	

M = Mean; SE = Standard error; VI = Visual impairment; HI = Hearing impairment; MD = Masticatory discomfort.

^†^Bonferroni correction; a,b,c: the presence of the same letters indicate a non-significant difference.

**Table 2.** Differences in Quality of Life according to the Coexistence of Visual Impairment, Hearing Impairment, and Masticatory Discomfort (N = 1,489)

Variables			n (%)	M ± SE	t (*p*)
A	VI only	Yes	169 (16.3)	0.98 ± 0.00	0.35 (.724)
		No	807 (83.7)	0.98 ± 0.00	
	HI only	Yes	123 (12.7)	0.97 ± 0.01	-1.49 (.138)
		No	807 (87.3)	0.98 ± 0.00	
	MD only	Yes	238 (22.5)	0.96 ± 0.01	-2.12 (.035)
		No	807 (77.5)	0.98 ± 0.00	
B	VI and HI	Yes	29 (3.3)	0.89 ± 0.03	-2.63 (.009)
		No	807 (96.7)	0.98 ± 0.00	
	VI and MD	Yes	57 (5.9)	0.93 ± 0.02	-2.65 (.009)
		No	807 (94.1)	0.98 ± 0.00	
	HI and MD	Yes	50 (4.3)	0.94 ± 0.02	-2.00 (.047)
		No	807 (95.7)	0.98 ± 0.00	
C	VI, HI, and MD	Yes	16 (1.5)	0.88 ± 0.04	-2.68 (.008)
		No	807 (98.5)	0.98 ± 0.00	

M = Mean; SE = Standard error; VI = Visual impairment; HI = Hearing impairment; MD = Masticatory discomfort.

**Table 3.** Influence of the Coexistence of Visual Impairment, Hearing Impairment, and Masticatory Discomfort on Quality of Life

Variables		Model 1^†^			Model 2^‡^			Model 3^§^			Model 4^∥^		
		β	t	*p*	β	t	*p*	β	t	*p*	β	t	*p*
A	VI only	0.00	0.35	.724	0.01	1.04	.301	0.01	0.92	.358	0.01	1.10	.273
	HI only	-0.01	-1.49	.138	-0.01	-1.45	.148	-0.01	-1.61	.109	-0.01	-1.47	.142
	MD only	-0.01	-2.12	.035	-0.01	-1.63	.105	-0.01	-1.37	.172	-0.01	-1.16	.247
B	HI and MD	-0.03	-2.00	.047	-0.03	-1.52	.131	-0.02	-1.21	.226	-0.02	-1.09	.276
	VI and MD	-0.05	-2.65	.009	-0.04	-2.50	.013	-0.04	-2.47	.014	-0.04	-2.47	.014
	VI and HI	-0.09	-2.63	.009	-0.09	-2.61	.010	-0.09	-2.77	.006	-0.09	-2.65	.009
C	VI, HI, and MD	-0.10	-2.68	.008	-0.09	-2.39	.018	-0.08	-2.02	.029	-0.09	-2.30	.022
R²		3.0%			5.6%			7.3%			8.8%		
Wald F		7.19			5.70			4.85			5.28		
*p*		.008			.018			.029			.022		

VI = Visual impairment; HI = Hearing impairment; MD = Masticatory discomfort.

^†^none; ^‡^sex, education level; ^§^sex, education level, drinking frequency, smoking status, physical activity, obesity status; ^∥^sex, education level, drinking frequency, smoking status, physical activity, obesity status, hypertension, dyslipidemia, stroke, cardiovascular disease, diabetes mellitus.

## Data Availability

The data that support the findings of this study are available from the corresponding author upon reasonable request.
